# Fucoidan Fractionated from *Sargassum coreanum* via Step-Gradient Ethanol Precipitation Indicate Promising UVB-Protective Effects in Human Keratinocytes

**DOI:** 10.3390/antiox10030347

**Published:** 2021-02-26

**Authors:** Ilekuttige Priyan Shanura Fernando, Mawalle Kankanamge Hasitha Madhawa Dias, Dissanayaka Mudiyanselage Dinesh Madusanka, Eui Jeong Han, Min Ju Kim, Soo-Jin Heo, Ginnae Ahn

**Affiliations:** 1 Department of Marine Bio-Food Sciences, Chonnam National University, Yeosu 59626, Korea; Shanura@chonnam.ac.kr; 2 Department of Food Technology and Nutrition, Chonnam National University, Yeosu 59626, Korea; 198807@jnu.ac.kr (M.K.H.M.D.); 198793@jnu.ac.kr (D.M.D.M.); iosu5772@naver.com (E.J.H.); alswn1281@nate.com (M.J.K.); 3 Jeju International Marine Science Center for Research & Education, Korea Institute of Ocean Science & Technology (KIOST), Jeju 63349, Korea; sjheo@kiost.ac.kr

**Keywords:** fucoidan, *Sargassum coreanum*, UVB-protection, oxidative stress, step-gradient ethanol precipitation

## Abstract

Fucoidans exhibit a wide range of bioactivities and receive significant attention in functional food and cosmetic research. Industrial applications of fucoidan are limited partially due to high extraction and purification costs. The present study implements an enzyme-assisted extraction and step-gradient ethanol precipitation for fractionating fucoidan from *Sargassum coreanum* based on its charge and molecular weight and evaluation of ultraviolet B (UVB) protective effects in human keratinocytes (HaCaT). The fucoidan fraction SCOC4 indicated higher fucose and sulfate contents with Fourier-transform infrared and ^1^H NMR spectral patterns resembling fucoidans. SCOC4 dose-dependently abated UVB-induced keratinocyte damage via suppressing intracellular reactive oxygen species, apoptotic body formation, DNA damage via suppressing mitochondria-mediated apoptosis. UVB-protective effects of SCOC4 were further attributable to the augmentation of nuclear factor erythroid 2-related factor 2 mediated cellular antioxidant defense enzymes. Step-gradient ethanol precipitation was a convenient approach of fractionating fucoidans based on molecular weight and charge (depend on the degree of sulfation). Further evaluation of seasonal variations, biocompatibility parameters, efficacy, and shelf life may widen the use of *S. coreanum* fucoidans in developing UVB-protective cosmetics and functional foods.

## 1. Introduction

The skin is the most superficial organ of the body and has vital physiological barrier functions protecting internal tissues from harmful external factors. Furthermore, the attractiveness of skin serves as a communal interface providing a perception of age, health, and social stratification [1]. Therefore, in the field of dermatology, a growing interest is paid towards maintaining skin health and its appealing appearance. Skin aging could be both intrinsic (chronological aging) or extrinsic caused by exposure to ultraviolet (UV) radiation (photoaging) and xenobiotics [2]. Chronic symptoms of photoaging include laxity, roughness, thickness, pigmentation, and wrinkling, and extreme conditions that can cause skin cancer.

The concept of “cosmeceuticals”, the use of natural products as functional ingredients of cosmetics, has recently emerged as a topic of great interest owing to health beneficial effects elicited by natural products apart of their intended activities [3]. Brown seaweeds are a source of polysaccharides such as alginate, fucoidan, and laminarin that have applications in industrial biotechnology. Fucoidan is a brown alga-derived fucose-rich sulfated polysaccharide well known for multifunctional bioactivities. The backbone of fucoidan is constructed of randomly sulfated 1→3 or 1→4 linked α-l-fucopyranose units periodically and randomly interrupted by other monosaccharides [4]. Hence the structure of fucoidan is highly heterogenous with certain species-specific similarities in low molecular weight (MW) fractions. UV-protective effects of fucoidans sourced from numerous seaweeds are reported in recent studies. Low MW fucoidans prepared by hydrolyzing high MW fucoidan by the enzyme fucoidanase from *Pseudoalteromonas* sp. (strain 1493) has shown protective effects against oxidative stress, matrix metallopeptidases expression, inflammation, wrinkle formation, and apoptosis in the UVB-irradiated mouse model [1]. *Hizikia fusiforme* derived fucoidans inhibit UVB-induced reactive oxygen species (ROS) levels in keratinocytes and zebrafish [5]. Molecular evidence suggests that fucoidans are activators of nuclear factor erythroid 2-related factor-2 (Nrf2) mediated antioxidant signaling that up-regulates downstream target genes, including heme oxygenase-1 (HO-1), NAD(P)H dehydrogenase [quinone] 1 (NQO1), glutathione peroxidase 1 (GPX1), and superoxide dismutase (SOD)1 and 2 [6].

*Sargassum coreanum* that abundantly grows in the coastal waters of Jeju Island, Korea is among the seaweed used to make Momguk a customary soup in Jeju Island. The functional values of *S. coreanum* have been barely explored where it could be an extremely sustainable resource of bioactive natural products [7,8]. Two previous studies report the antioxidant activity of Celluclast-assisted extracts of *S. coreanum* and its subsequent fractions obtained via ultrafiltration [7,9]. However, the active components responsible for bioactivity were not identified. The present study was undertaken as a part of a project investigating the bioactive effects of fucoidans sourced from abundant brown seaweeds of South Korea to promote its industrial biotechnology applications. Based on the aforementioned, the present study hypothesizes UVB-protective effects of fucoidans derived from *S. coreanum* in abating oxidative stress and apoptosis in keratinocytes. This work aimed to develop an environmentally friendly and efficient method for extraction of fucoidan from *S. coreanum* and to establish step-gradient ethanol precipitation as a convenient method for further purifying fucoidans.

## 2. Materials and Methods

### 2.1. Materials

Deuterium oxide, 2′ 7′-dichlorodihydrofluorescein diacetate (DCFH2-DA), Fucoidan standard, 3-(4,5-dimethylthiazol-2-yl)-2,5-diphenyltetrazolium bromide (MTT), propidium iodide (PI), acridine orange, ethidium bromide, 2-mercaptoethanol, o-Toluidine blue, trifluoroacetic acid, Hoechst 33342, and sodium dodecyl sulfate were bought from Sigma-Aldrich Co (St. Louis, MO, USA). Celluclast was obtained from Novozyme (Bagsvaerd, Denmark). Polyvinylidene fluoride membranes were obtained from Millipore (Schwalbach, Germany). Analytical grade solvents chloroform, methanol, and ethanol were purchased from Daejung Chemicals & Metals (Gyeonggi-do, Korea). Dulbecco’s Modified Eagle Medium (DMEM), Fetal Bovine Serum (FBS), and penicillin/streptomycin mixture were acquired from GIBCO INC. (Grand Island, NY, USA). Antibodies for western blot analysis were purchased from Cell Signaling Technology (Beverly, MA, USA) and Santa Cruz Biotechnology, Inc. (Santa Cruz, CA, USA).

### 2.2. Extraction of S. coreanum

*S. coreanum* coarse powder purchased by Paraje Co., Ltd., Jeju-do, Korea, was pulverized using an IKA MF 10 basic pulverizer (Werke, Germany). Algae powder was first depigmented using a mixture of chloroform and methanol 1:1. The power was left suspended in a 10% formaldehyde/ethanol solution at 40 °C for 5 h. Afterward, the powder was repeatedly washed with 80% ethanol to remove remains of formaldehyde. After pre-treatment, the powder was desiccated at 50 °C in a forced convection oven. The powder was suspended in deionized water, and the pH of the suspension was adjusted to pH 5.0 with 1 M HCl while incubating at 50 °C. Although observing no change in the pH, Celluclast was introduced to the suspension at a 0.5% ratio, and the incubation was continued for 8 h under moderate agitation. After enzymatic digestion, the suspension was first filtered by a stainless steel extra fine mesh strainer, and the filtrate was centrifuged (5000× *g*) to remove undissolved particles. The supernatant was incubated for 10 min in a boiling water bath to deactivate Celluclast. Next, CaCl_2_ (2% final concentration) was added to the filtrate and left at 4 °C for 3 h. Again, the mixture was centrifuged (5000× *g*) to remove calcium alginate. The pH of the mixture was adjusted to 7.0 by adding 1M NaOH, and the extract was partially lyophilized, concentrating it to 1/4th of its initial volume.

### 2.3. Step-Gradient Ethanol Precipitation

The extract was pre-cooled at 4 °C while moderately mixing with 250 mL of cold ethanol to obtain the first precipitate. The mixture was kept under moderate agitation at 4 °C for 12 h for equilibration. The initial precipitate was recovered by centrifuging the mixture at 5000× *g*. Each subsequent precipitate was obtained by step wisely, adding ethanol (500 mL, 1 L, and 2 L) to the supernatant of the former fraction, equilibrating each at 4 °C for 12 h. Each precipitate obtained was repeatedly washed with 95% ethanol until no significant color change is observed. Finally, each of the precipitate was dissolved in deionized water, and freeze-dried, obtaining polysaccharide powder.

### 2.4. Analysis of Fractionation Efficiency by Agarose Gel Electrophoresis

The estimated MW, homogeneity, and fractionation efficiency of polysaccharide fractions were analyzed by agarose gel electrophoresis [10]. Briefly, 1 mg mL^−1^ of MW standards and fractionated polysaccharides were loaded and separated on 1% agarose gels by electrophoresis at 100 V for 20 min. Finally, the gel was stained by dipping in toluidine blue and 3% acetic acid solution for 1 h, which was de-stained using 3% acetic acid.

### 2.5. Fourier-Transform Infrared (FTIR) Analysis

FTIR spectra of powdered samples were obtained by a PerkinElmer, Spectrum 400 system equipped with a universal attenuated total reflectance accessory (Waltham, MA, USA). Spectra were collected within the 4000 to 500 cm^−1^ wavenumber range at a 4 cm^−1^ spectral resolution, with 32 co-added scans. FTIR spectra were analyzed by PerkinElmer Spectrum software and graphed using OriginPro 8 software.

### 2.6. Composition Analysis of Monosaccharides

SCOC4, which indicated the best bioactivity, was analyzed for its monosaccharide composition by Carbohydrate Bioproduct Research Center, Sejong University, South Korea. Briefly, SCOC4 was hydrolyzed using trifluoroacetic acid at 105 °C, and the acid was evaporated under a vacuum. The sample was diluted (0.02%) and resolved using a high-performance anion-exchange chromatography with pulsed amperometric detection (HPAEC-PAD) system with a CarboPac ™ PA1 column intergraded to a Dionex ED50 Detector. Monosaccharides were identified and quantified by comparing with retention times of standard curves.

### 2.7. ^1^H NMR Analysis

^1^H NMR analysis was done on a JNM-ECX 400 NMR spectrometer (JEOL, Tokyo, Japan) at 25 °C. SCOC4 35 mg was deuterium exchanged with repeated deuterium oxide solvation and lyophilization and used in the analysis by dissolving in deuterium oxide. Spectra were obtained by Delta software version 4.3.6 and analyzed by MestReNova software.

### 2.8. Cell Culture

#### 2.8.1. Maintenance of Cells

HaCaT human keratinocytes were obtained by the Korean Cell Line Bank (Seoul, Korea). Cell cultures were maintained in DMEM media supplemented with 10% of FBS and 1% of penicillin/streptomycin mixture. Sub-culturing was done once every two days. Exponentially growing cells were seeded for experiments. After 24 h of incubation in multi-well plates, different sample concentrations were treated and incubated for 24 h. Cell viability was examined by MTT assay [10]. Absorbance was measured by a Molecular Devices, SpectraMax M2 microplate reader (Sunnyvale, CA, USA).

#### 2.8.2. UVB Exposure and Intracellular ROS Level Analysis

Preliminary dose and time response studies were carried out to optimize conditions for experiments. HaCaT cells (1 × 10^5^ cells mL^−1^) were seeded in a 24 well plate for 24 h and treated with 25, 50, 100, and 200 µg mL^−1^ of sample concentrations for 2 h. The culture medium was replaced by phosphate-buffered saline (PBS) before the UVB exposure. Wells corresponding to the control group was covered with foil, and the rest of the plate was exposed to 50 mJ cm^−2^ of UVB using a UVP CL-1000L ultraviolet cross-linker (Upland, CA, USA). Immediately PBS was removed, and the wells were washed and replaced by serum-free culture media. Cells were incubated for 1 h for determining ROS levels by the DCFH2-DA method [11]. However, for the evaluation of cell viability (MTT assay), the cells were incubated for 24 h after the UVB exposure [12]. 

Further experiments were conducted using SCOC4, which indicated better cytoprotective and antioxidant effects compared to other fractions. Accordingly, DCFH2-DA stained HaCaT cells following sample treatment and UV exposure were visualized under a Thermo Fisher Scientific, EVOS FL Auto 2 Imaging microscope (Rockford, IL, USA), and Beckman Coulter CytoFLEX flow cytometric analysis (Coulter, PA, USA). The methods are mentioned in our previous publication [12].

#### 2.8.3. Nuclear Staining with Hoechst 33342 and Acridine Orange/Ethidium Bromide Double Staining

The experimental protocols for nuclear staining are outlined in our previous publication [13]. Briefly, the cells after treatment and UV exposure were incubated for 24 h. The well plates were treated with Hoechst 33342 or a mixture of acridine orange and ethidium bromide and incubated for 10 min in the dark. Stained cells were observed on an EVOS FL Auto 2 Imaging microscope.

#### 2.8.4. Alkaline Comet Assay

Alkaline comet assay was carried out to assess the extent of DNA damage according to [13]. Briefly, the cells harvested after sample treatment and UVB exposure were suspended in low-melting agarose at 37 °C and gently applied on the surface of slides pre-coated with agarose. Cells were lysed and electrophoresed at 4 °C for 30 min applying 15 V. The comets were visualized by ethidium bromide staining on an EVOS FL Auto 2 Imaging microscope.

#### 2.8.5. Cell Cycle Analysis

The proportion of sub-G_1_ hypodiploid cells was determined by flow cytometry analyses [13]. Briefly, cells were PBS washed and permeabilized in 70% ethanol in PBS. After pelleting down, the cells were again resuspended and washed in a PBS/EDTA solution. Cells were resuspended in a PBS solution containing PI, EDTA, and RNase A for 10 min and washed with PBS/EDTA solution. Cells were analyzed on a Beckman Coulter CytoFLEX flow cytometer.

#### 2.8.6. Western Blot Analysis

Cells were lysed using a Thermo Scientific, NE-PER^®^ nuclear, and cytoplasmic extraction kit (Rockford, IL, USA). Protein (40 μg) was electrophoresed on 15% sodium dodecyl sulfate-polyacrylamide gels. Resolved proteins were transferred onto nitrocellulose membranes and blocked using 5% skim milk in TBST. Membranes were sequentially incubated with primary (1:1000) and HRP-conjugated secondary antibodies (1:3000). An ECL regent was applied to protein bands and detected on a Davinch-ChemiTM imaging system (Core Bio, Seoul, Korea) [14].

### 2.9. Statistical Analysis

All data are expressed as mean ± standard deviation of at least three determinants. Statistical comparisons were performed via one-way analysis of variance followed by Duncan’s multiple range test, using predictive analytics software (PASW) Statistics 19.0 (SPSS, Chicago, IL, USA). P values of less than 0.05 “*” and 0.01 “**” were considered significant.

## 3. Results

### 3.1. Yield and Proximate Composition of Polysaccharide Fractions

Celluclast extraction yield of *S. coreanum* was recorded as 27.14 ± 0.43% from the dry weight of algae. The extraction yield was highest (12.25%) in the fraction SCOC1 (Table 1). The polysaccharide and sulfate contents indicated an antiparallel relationship when going through subsequent fractions. The contents of polyphenol, protein, and ash were considerably low in every fraction.

### 3.2. Molecular Weights (MW) of Polysaccharide Fractions and Vibrational Spectra

**Figure 1:**
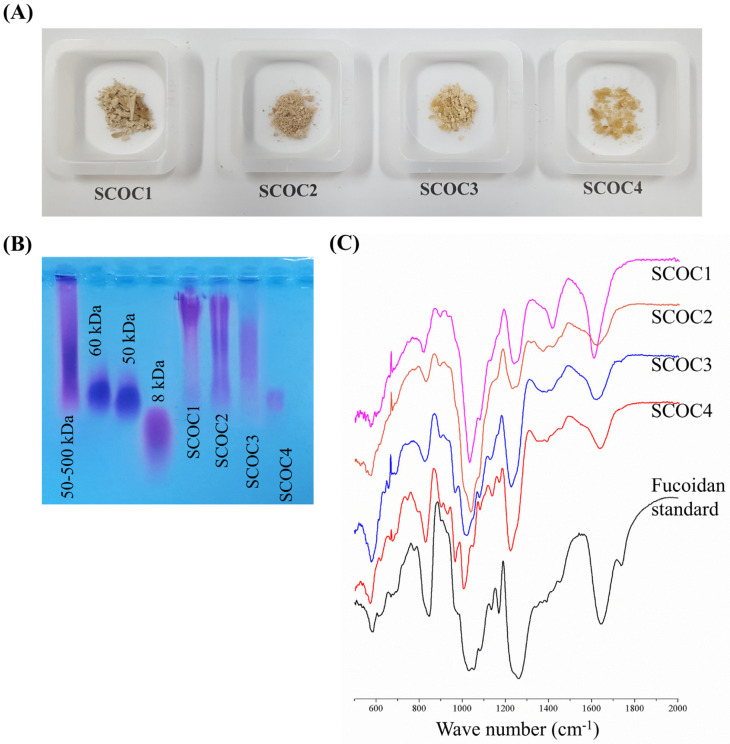
Characterizing molecular weights and FTIR spectra of different polysaccharide fractions refined by step-gradient ethanol precipitation. (**A**) The appearance of fucoidan fractions SCOC1, SCOC2, SCOC3, and SCOC4. Analysis of polysaccharide fractions for their (**B**) molecular weight distributions and (**C**) FTIR spectra.

The fucoidan fractions appeared in light brown (Figure 1A). As Figure 1B indicates, The MWs of fractions SCOC1-SCOC4 were narrowing down with SCOC4 being nearly 50 kDa. The difference in MW suggests a considerably good separation of polysaccharide fractions. Vibrational modes (Figure 1C) were assigned based on previous publications. The 840 cm^−1^ peak is attributable to the bending of axial sulfate (C-O-S) substituents in fucose units. The broadened peak (due to overlap of peaks) between 1200–970 cm^−1^ corresponds to stretching vibrations of glycosidic bonds and bonds in pyranoid rings (C-O and C-C). The prominent peak between 1220–1270 cm^−1^ is arising from stretching vibrations of S=O bond in sulfate groups. This peak is a unique identifier of sulfated polysaccharides. The 1620 cm^−1^ peak can be assigned to the bending vibration of O-H groups [15,16,17,18]. The height/intensity of 1220–1270 cm^−1^ peak increased going through subsequent fraction, which suggests an increase of sulfate groups.

### 3.3. Protective Effects of Fucoidans Against UVB-Induced Oxidative Stress

**Figure 2:**
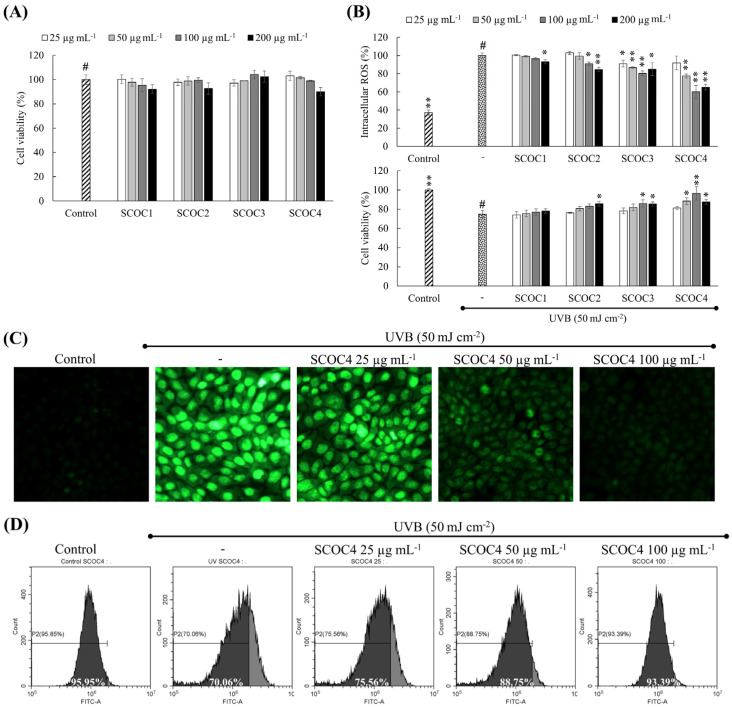
Cytotoxicity of polysaccharide fractions and their protective effects on HaCaT keratinocytes against UVB-induced oxidative damage. (**A**) Analyzing cytotoxicity doses of polysaccharide fractions. (**B**) Intracellular antioxidant and cytoprotective potential of polysaccharide fractions in UVB stimulated keratinocytes. Intracellular antioxidant activity of SCOC4 by (**C**) fluorescence microscopy and (**D**) flow cytometry analysis. For ROS assays, the cells were stained with DCFH2-DA. Values are reported as means ± SD of three independent determinations (*n* = 3). * *p* < 0.05 and ** *p* < 0.01 display significant differences compared with group indicated by “#”.

Preliminary dose-response cytotoxicity and ROS-inhibitory effects of fractions were estimated using sample concentrations 6.25, 12.5, 25, 50, 100, 200, and 400 µg mL^−1^ without replicates (data is not presented). Among them, the repeatability of the outcomes was confirmed for concentrations 25–200 µg mL^−1^ (Figure 2A). The dose-response analysis indicated that the concentrations 25–200 µg mL^−1^ are generally safe for treatment per the observation of >80% cell viability. As shown in Figure 2B a prompt increase of intracellular ROS level was apparent for UVB exposed cells, with a pronounced reduction of cell viability. The UVB exposure of 50 mJ cm^−2^ was chosen after preliminary analysis involving UVB exposure of 30, 40, 50, 60, 70, 80 90, and 100 mJ cm^−2^. The dose-response fluctuation of intracellular ROS levels and cell viability with different polysaccharide fractions indicated an interesting pattern where a prompt reduction of intracellular ROS levels was apparent in SCOC4 (25–100 µg mL^−1^) with an augmentation of cell viability. However, the 200 µg mL^−1^ concentration of SCOC4 indicated a cytotoxic response deviating from the trend seen for the 25–100 µg mL^−1^ range.

According to fluorescence microscopy, UVB exposure of HaCaT cells indicated an increased green fluorescence (Figure 2C). The corresponding rightward shift of cell population across the FITC-A axis in the flow cytometry histogram (Figure 2D) agrees with the increased fluorescence of cells. Intracellular ROS levels dose-dependently decreased with SCOC4 treatment, whereas a higher recovery was seen for 100 µg mL^−1^ concentration. The ROS probe DCFH2-DA used in the above experiment is a cell-permeable dye. Intracellular esterases convert DCFH2-DA to membrane-impermeable compound, H2DCF, that gets oxidized into DCF emitting green fluorescence [19].

### 3.4. Determination of Structural Properties of SCOC4 by ^1^H NMR and Monosaccharide Composition Analysis

**Figure 3:**
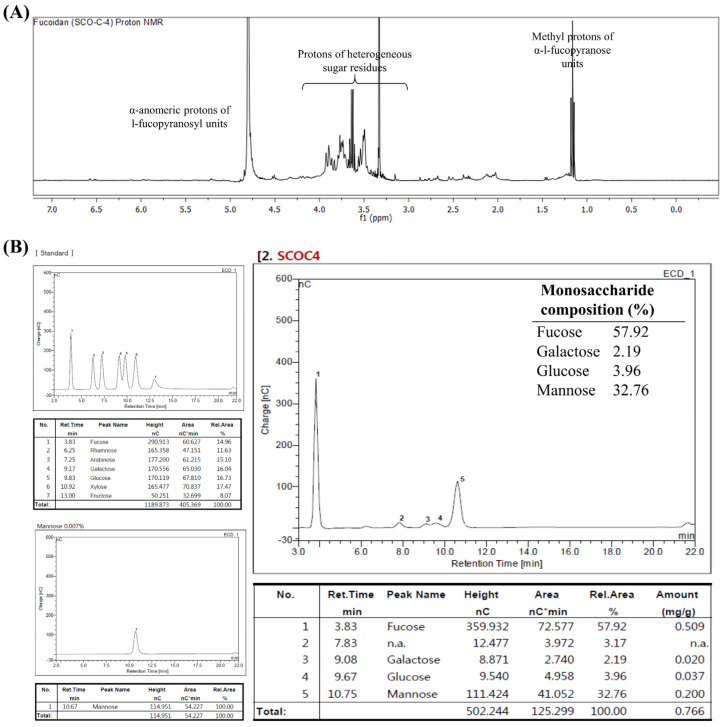
Characterizing structural properties of SCOC4. (**A**) ^1^H NMR spectrum and (**B**) monosaccharide composition of SCOC4.

The ^1^H NMR spectrum of SCOC4 (Figure 3A) was weak in resolution due to the heterogeneous nature and structural complexity of the polymer. However, the spectrum indicated unique patterns and peaks corresponding to fucoidans when comparing with data of previous publications [10,17,20,21]. Signals between 1.1 and 1.4 ppm are indicative of protons of methyl groups attached to α-L-Fucopyranose residues. Peaks between 3.0–4.3 ppm could be attributed to H2-H5 protons found in heterogeneous sugar residues. Peaks between 5.0–5.5 ppm region represent signals of anomeric protons [20,21]. Figure 3B indicates the composition of monosaccharides in SCOC4 composing of high fucose (57.92%) and mannose content (32.76%).

### 3.5. SCOC4 Reduced UVB-Induced Apoptotic Body Formation and DNA Damage

**Figure 4:**
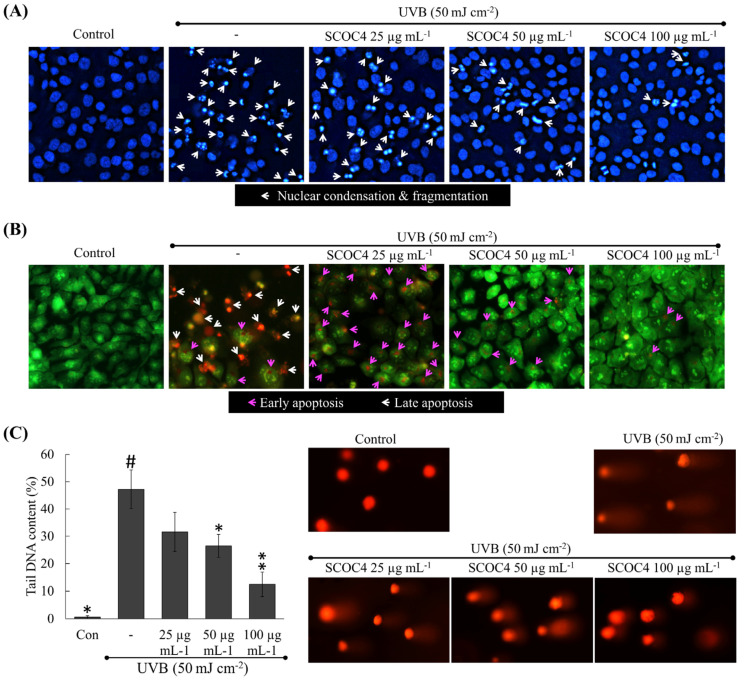
Protective effects of SCOC4 on suppression of UVB-induced apoptosis and DNA damage in HaCaT keratinocytes. Examining nuclear morphology by different staining assays involving (**A**) Hoechst 33342 and (**B**) acridine orange and ethidium bromide nuclear double staining. (**C**) Single-cell DNA damage analysis by comet assay in UVB-induced keratinocytes. Values are reported as means ± SD of three independent determinations (*n* = 3). * *p* < 0.05 and ** *p* < 0.01 display significant differences compared with group indicated by “#”.

According to Figure 4A, UVB exposure caused chromatin condensation as well as nuclear fragmentation, which are indicative of apoptotic bodies. Per the double staining (Figure 4B), the presence of orange-colored fragments indicates cells that are in late apoptosis. With SCOC4 treatment, a dose-dependent reduction of apoptotic bodies could be observed. The results were repeatable and confirmed that SCOC4 treatment causes a reduction of nuclear membrane damage, which is the cause of observing late apoptotic cells. Cellular DNA damage is another distinctive indication of apoptosis, which could be assessed by comet assay. As indicated in Figure 4C, the tail length and area corresponding to DNA content were highest in UVB stimulated cells signifying increased DNA damage. The tail DNA content dose-dependently decreased following SCOC4 treatment attributable to UVB-protective potential of SCOC4.

### 3.6. SCOC4 Suppressed UVB-Induced Accumulation of Sub-G_1_ Hyperdiploid and Apoptotic Cells

**Figure 5:**
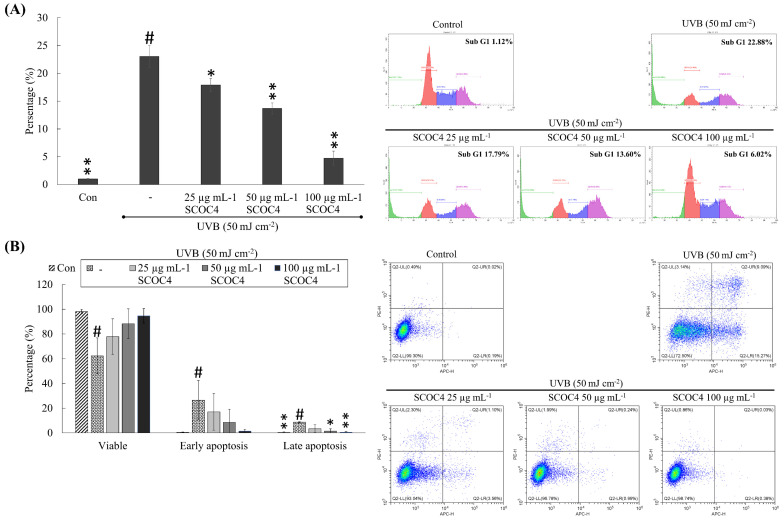
The protective role of SCOC4 upon the migration of UVB stimulated cells into apoptosis. The effect of SCOC4 on UVB-induced (**A**) accumulation of sub-G_1_ apoptotic populations by flow cytometry (PI stain) and (**B**) migration of cells into early and late phases of apoptosis by annexin V assay. Values are reported as means ± SD of three independent determinations (*n* = 3). * *p* < 0.05 and ** *p* < 0.01 display significant differences compared with group indicated by “#”.

Based on cell cycle analysis (Figure 5A), UVB exposure caused a prompt increase in the sub-G_1_ apoptotic cells (23.05 ± 1.98%) compared to the control (1.00 ± 0.11%). SCOC4 treatment dose-dependently reduced the sub-G_1_ cell population, indicating protective effects parallel to previous experimental outcomes. Flow cytometric analysis with annexin V was conducted to monitor the migration of cells to the early stage of apoptosis. For the assay, cells were harvested 6 h after the UVB stimulation. As indicated in Figure 5B, 21.51 ± 8.82% of cells migrate into early and 8.69 ± 0.57% into the late stage of apoptosis following 6 h of UVB exposure. However, SCOC4 pre-treated cells indicated a dose-dependent reduction of UVB-induced apoptosis.

### 3.7. SCOC4 Decreased Cytochrome C Release and Mitochondria-Mediated Apoptosis in UVB-Irradiated Cells

**Figure 6:**
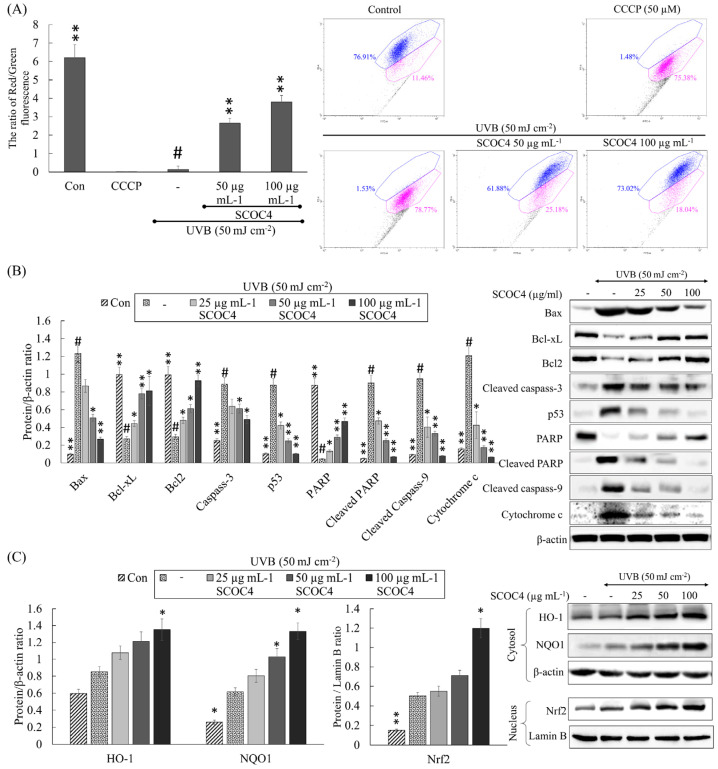
Role of SCOC4 on suppressing mitochondria-mediated apoptosis in UVB stimulated HaCaT keratinocytes. The effect of SCOC4 on UVB-induced (**A**) mitochondrial damage by JC-1 assay. Inhibitory effects of SCOC4 on (**B**) major markers of mitochondria-mediated apoptosis and (**C**) Nrf2-mediated intracellular antioxidant enzymes in UVB-induced HaCaT keratinocytes. Values are reported as means ± SD of three independent determinations (*n* = 3). * *p* < 0.05 and ** *p* < 0.01 display significant differences compared with group indicated by “#”.

Mitochondria are pivotal in orchestrating biochemical execution of cells through apoptosis by interrelated control of electron transport, energy metabolism, and release of caspase activators [22]. Numerous signals that converge on mitochondria triggering or inhibiting the above events and subsequent downstream effects, delineate key pathways of physiological cell death. Mitochondrial apoptosis is initiated due to the alteration of its inner transmembrane potential (ΔΨm), which causes permeability transition pores to be opened, causing the discharge of caspase activators, which induces apoptosis. Flow cytometry JC-1 assay allows the highly specific evaluation of mitochondrial health based on the differential accumulation of JC-1 dye. Based on present outcomes (Figure 6A), UVB exposed cells indicated a higher proportion of mitochondrial damage closely similar to cells treated with the commercial mitochondrial membrane-potential disrupter, carbonyl cyanide m-chlorophenyl hydrazine (CCCP). Treatment of SCOC4 dose-dependently attenuated the proportion of cells with UVB-induced mitochondrial damage indicating its protective effects.

Western blot analysis indicated (Figure 6B) an outflow of cytochrome c following UVB irradiation. The above evidence is in line with the increase of Bcl-2-associated X protein (Bax), caspase-3, cleaved caspase-9, and p53 levels, and poly (ADP-ribose) polymerase (PARP) cleavage together with inhibition of anti-apoptotic proteins, Bcl2 and Bcl-xL. SCOC4 treatment dose-dependently ameliorated the alteration of molecular mediators mentioned above. Heme oxygenase (HO-1) is one of the key enzymes that suppress cellular oxidative stress. Nrf2 is a multifunctional regulator of numerous genes associated with antioxidant and anti-inflammatory activity, including key enzymes HO-1, glutamate-cysteine ligase, thioredoxin reductase 1, and NAD(P)H-quinone oxidoreductase 1 [23]. Activation of Nrf2 provides robust protection against oxidative stress. Based on western blot results, UVB exposure slightly increased Nrf2 HO-1 and NQO1 levels (Figure 6C). Treatment of SCOC4 further increased the Nrf2 and HO-1 levels revealing its stimulatory effects.

## 4. Discussion

The natural product-based cosmetic industry has recently acquired an increased market value concerning the robust properties of natural ingredients over synthetic agents. At present, studies are conducted to explore the bioactive potential of marine bioresources as a sustainable source for producing cosmeceuticals and nutricosmetics. Many recent studies confer the wide-ranging bioactivities of seaweed fucoidans. Cytoprotective effects elicited by fucoidan are primarily mediated via suppressing the cellular oxidative stress [4,21,23,24].

Crude fucoidan for industrial manufacturing processes is obtained conventionally by hot water (40–60 °C) extraction of powdered seaweed. CaCl_2_ is added to the extracts to precipitate alginate, and fucoidan is recovered by adding large volumes of ethanol [10,18,25]. Recent studies show that enzymatic degradation of algal cell walls using food-grade commercial cellulases gives a higher extraction yield over conventional methods while ensuring their safety for numerous applications [10]. Celluclast is a desirable enzyme in this regard. 

Chloroform and methanol (1:1) pre-treatment eliminate lipophilic contaminants increasing the hydrophilicity of the algal powder [25]. The agitation of sample powder in a mixture of formaldehyde (10%) in ethanol helps to reduce the co-extraction of polyphenolic compounds. Herein formaldehyde polymerizes polyphenols, making them less soluble in the extracting solvent [25]. The effectiveness of the present protocol is reflected by the reduced contents of polyphenols observed in all fractions (Table 1). The optimum pH (4.5) and temperature used during Celluclast-assisted extraction makes it ideal for reducing alginate contamination while increasing the extraction efficiency. At pH 4.5 majority of water-soluble alginates convert to water-insoluble alginic acid and thereby remain without dissolving in the extraction solvent. Disregarding the pre-treatment, the Celluclast extraction yield, 27.14 ± 0.43% is acceptable compared to the yields recorded from water extracts of other *Sargassum* species [18]. 

As per industrial manufacturing, fucoidan is recovered from the extract by prompt reduction of dielectric constant, which is subsequently fractionated by employing techniques such as membrane filtration or size exchange chromatography [26]. Fucoidans refined by conventional methods may comprise a heterogeneous mix of complex polymer chains. Here we utilize step-gradient ethanol precipitation, a convenient and efficient technique to fractionate polysaccharides depending on their solubility that relies on their MW [27]. This is a cost-effective method of reducing the heterogeneity of fucoidans. The outcomes of MW distribution analysis reflect the effectiveness of step-gradient ethanol precipitation in fractionating fucoidan. As an efficient and cost-effective method, step-gradient ethanol precipitation has extensive industrial applications in fractionating starch, hemicellulose, glucan, pectin, arabinan, fructan, and pullulan [27]. 

The structural complexity of fucoidan is defined by the MW of polysaccharides, the composition of monosaccharides, connectivity, and sulfate content. Per present observations, subsequent fractions obtained via gradient precipitation had gradually increasing sulfate and fucose contents with a decrease of average MW. UVB-protective effects of fractions indicated superlative activity for SCOC4 compared to other fractions. The bioactivity relation of fucoidan with the degree of sulfation, fucose content, and MW has been demonstrated previously by several studies [1,28]. Accordingly, fucoidans with a higher degree of sulfation and lower MW possess better antioxidant, anti-inflammatory, and anti-aging activities.

UVB can penetrate the stratum corneum, causing ROS production in underlying cells, initiating a cascade of cellular responses ultimately leading to apoptosis. These events cause the pathogenesis of numerous symptoms, including an increase of epidermis thickness, discoloration, loss of elasticity, and retard skin cell growth rate [7]. Numerous UVB-protective cosmetic formulations are available in the market. However, their chronic usage may cause side effects in the long run. The use of natural cosmetics as UV-protective agents is long acknowledged and is considered robust compared to synthetic material. SCOC4 treatment from 25–100 µg mL^−1^ promptly reduced the intracellular ROS levels in UVB exposed cells. The increase of intracellular ROS level and reduction of cell viability per 200 µg mL^−1^ SCOC4 could be rationalized by considering the immunomodulatory properties of sulfated polysaccharides [26]. Present observations on UVB-protective effects suggested that SCOC4 is effective at 25–100 µg mL^−1^ dose range. Low MW fucoidans (≈0.8 KDa) from *Sargassum hemiphyllum* that have a sulfate content of 38.9 ± 0.4 % have indicated UVB-protective effects at 100 µg mL^−1^ in Hs68 human foreskin fibroblasts [29]. Fucoidan purified from *H. fusiforme* that has an MW of 102.67 kDa have its UVB-protective effects at 12.5–50 µg mL^−1^ dose range [30]. Hence, the UVB-protective effects of SCOC4 range within generic doses reported for fucoidans. Algal natural products such as polyphenols (phenolic acids, phlorotannins, flavonoids) are renowned for their antioxidant effects at doses as lower as 10 μM [31]. However, higher doses of polyphenols are associated with cytotoxic effects and should therefore be used under a specific dose range. In this regard, fucoidan, which is approved by Food and Drug Administration (FDA) is a safe substance classified under the Generally Recognized As Safe (GRAS) category offers advantages for use as ingredients in cosmetic formulations [32].

Heterogenous fucoidan fractions are generally characterized by FTIR, ^1^H NMR, monosaccharide composition, and weight average MW [4,28]. Due to the complex connectivity and heterogeneous nature of fucoidans, the analysis of monosaccharide sequence and sulfation pattern remains highly challenging. In previous studies, methylation analysis of connectivity has been implemented in less heterogenous fucoidans after extensive purification [26]. Vibrational spectra of all fractions show unique spectral features, indicating fucoidan structure. The intensity of the peak 1220–1270 cm^−1^ corresponding to stretching vibrations of S=O bond in sulfate groups gradually increased in subsequent fractions. The above observation was in a consistent argument with the results observed for sulfate contents per chemical assay. The fucose content of SCOC4 was recorded as 57.92% with a 20.01 ± 0.04% of sulfate content. ^1^H NMR of SCOC4 indicated a close resemblance to fucoidans reported previously [10,17,20,21]. The present study does not provide information regarding the connectivity of monosaccharides in the obtained fucoidan fraction. Hence, bioassay-guided further purification of SCOC4 is necessary for simplifying the heterogeneity of fucoidans in the fractions. Additional experiments for methylation analysis involving chemical derivatization (methylation), desulfation, hydrolysis, reduction, acetylation, and GC-MS/MS analysis are required to obtain details on the monosaccharide connectivity pattern and substitution of sulfate groups [26]. Linkage analysis of fucoidans purified from *S. coreanum* has not yet been attempted. Per literature fucoidans found in other *Sargassum* species are mainly formed of (1 → 3) and (1 → 4)-linked -α-L-fucopyranose residues with sulfate groups located mainly on C2 of fucose and galactose units [33,34]. However, fucoidans being extremely complex heteropolysaccharides large deviations in the structure are reported for the connectivity of other monosaccharide units in different species of *Sargassum*.

Fluorescence microscopy and flow cytometry analysis were conducted to confirm the antioxidant potential of SCOC4 in suppressing UVB-induced intracellular ROS generation. Herein, flow cytometric analysis provides better outcomes over other ROS quantification methods as it eliminates fluorescence coming off from cellular debris. However, the provision of identical incubation conditions is crucial to minimize time, temperature, and light-dependent fluctuations of DCFH2-DA fluorescence intensity. Incubating tubes in ice during flow cytometry analysis increases the precision of the test trials. The dose-dependent reduction of intracellular ROS levels per different analysis methods demonstrates the antioxidant potential of SCOC4.

As firmly established by many studies, apoptosis resulting from oxidative stress is regulated via the intrinsic (mitochondria-mediated) pathway [35]. A variety of key events, including alteration of cellular oxidation-reduction balance signals the mitochondria to trigger several major pathways of physiological cell death [22]. The collapses of mitochondrial inner transmembrane potential (ΔΨm) cause the opening of mitochondrial permeability transition pores, causing an osmotic disequilibrium, matrix swelling, and rupture of the outer membrane leading to the release of cytochrome c and other caspase-activating proteins. Bcl-2 prevents the opening of permeability transition pores, whereas Bax and elevation of cytosolic Ca^2+^ levels activate its opening.

Cytochrome c binding to Apaf-1 results in the formation of apoptosome that activates downstream effector caspases initiating a proteolytic cascade culminating with apoptosis [13]. Ample evidence describes a pivotal role play by p53 in mediating apoptosis triggered by oxidative stress [36]. Activation of p53 causes PARP cleavage resulting in the deregulation of DNA stability. Per present observations, UVB exposure caused a prompt increase of mediators that trigger and propagate apoptosis while reducing anti-apoptotic factors such as Bcl2 and Bcl-xL. The altered levels of the aforementioned molecular mediators were ameliorated with SCOC4 treatment. Collectively, the observations of the present study suggest the protective ability of a low MW fucoidan fraction (SCOC4) in inhibiting apoptosis stimulated by UVB exposure.

Intracellular redox homeostasis is maintained by a complicated system of antioxidant enzymes that are implicated in protecting cells from oxidative stress. The redox-sensitive transcription factor, Nrf2, plays a pivotal role in this manner. Under physiological conditions, Nrf2 remains in the cytoplasm in an inactive state attached to Kelch-like-ECH-associated protein (Keap1). Upon activation, Nrf2 dissociates from Keap1 and gets translocated into the nucleus, interacting with antioxidant response elements. The translocation causes the subsequent expression of several downstream genes, which encode antioxidant enzymes, including HO-1, NQO1, and glutamate-cysteine ligase restoring redox homeostasis [37]. Nrf2 nuclear translocation was dose-dependently increased in keratinocytes following SCOC4 treatment and was higher compared to the control and UVB exposed cells. Both HO-1 and NQO1 levels increased in line with Nrf2 nuclear translocation. The results suggest that SCOC4 activates Nrf2 mediated expression of downstream antioxidant genes, which is responsible for protecting the cells from UVB-induced oxidative stress. As there could be a possibility that the components in FBS used in culture media such as steroids may synergistically influence the bioactivity, further experiments using culture media containing different doses of FBS may assist to resolve this presumption. When comparing SCOC4 with the mannofucan previously purified from *Sargassum horneri*, SCOC4 contained less fucose and indicated the presence of mannose, glucose, and galactose. The distribution of MWs of the fractions and their yields indicated a substantial deviation from each other. However, UVB-protective effects of both fucoidans were within the same range [38].

Topical application of UVB-protective drugs is a convenient way of delivering them to viable skin cell layers. However, the stratum corneum of the skin prevents the entry of compounds that are of higher MW over 350 Da. The relatively large MW, charge, and hydrophilicity of fucoidans minimize their skin penetration ability. This can be overcome by regulating the ointment formulation by incorporating penetration enhancers such as transcutol and surfactants such as polyethylene glycol [39]. The aforementioned ointment containing 15% of fucoidan with an average MW of 750 kDa has shown promising anti-Xa activity upon topical application in rats at 50–150 mg/g dose. Based on the present in vitro analysis, the requirement of a higher SCOC4 dose for achieving the desired bioactivity is a drawback in developing skincare products. Therefore, further studies using in vivo models are required to gain a satisfactory understanding of its effective dose requirements. Low MW fucoidans obtained by hydrolyzing high MW fucoidans via fucoidanase from *Pseudoalteromonas* sp. (strain 1493) are reported to be effective at 2.0 mg cm^−2^ [1]. Therefore, further hydrolysis of SCOC4 using fucoidanase can be implemented as a strategy to enhance its bioactivity.

## 5. Conclusions

Fucoidans purified from brown algae have recently received attention in functional food and cosmetic research per their wide-ranging health beneficial properties. The massive inundations of pelagic *Sargassum* in many parts of the world have recently provided wide opportunities for purifying fucoidan in scaled-up proportions. However, industrial use of fucoidan is limited partially due to its high extraction and purification costs. The present study employed an effective method for extracting fucoidans from *S. coreanum* using food-grade enzyme Celluclast followed by the fractionation via step-gradient ethanol precipitation. The fractions were screened for protective effects that they may exert against UVB exposure in HaCaT keratinocytes. Based on the outcomes, gradient ethanol precipitation enabled the fractionation of fucoidans based on their charge and MW. The present methodology holds significant advantages as an initial fractionation technique for fucoidans over conventional microfiltration and chromatographic purification techniques for refining low MW fucoidans. Considering the vast abundance of *S. coreanum*, extensive investigations may benefit its possible industrial use as raw material for purifying fucoidan and other ingredients. Based on present outcomes, *S. coreanum* derived low MW fucoidans indicated potent antioxidant properties that account for its UVB-protective effects. Extensive studies are required for evaluating its therapeutic effects on in vivo models followed by pharmacokinetic and clinical studies.

## Figures and Tables

**Table 1:** Yield and proximate composition of polysaccharide fractions.

Composition	SCOC1	SCOC2	SCOC3	SCOC4
**Yield (%)**	**12.25**	**6.90**	**1.81**	**1.18**
Polysaccharide (%)	64.70 ± 0.36	55.33 ± 0.36	52.05 ± 0.30	45.96 ± 0.36
Sulfate (%)	7.90 ± 0.02	12.15 ± 0.32	15.67 ± 1.20	20.01 ± 0.04
Polyphenol content (%)	1.42 ± 0.02	0.78 ± 0.15	0.56 ± 0.28	0.65 ± 0.16
Protein content (%)	1.00 ± 0.11	1.00 ± 0.18	0.85 ± 0.17	0.79 ± 0.06
Ash content (%)	1.03 ± 0.02	0.90 ± 0.00	0.76 ± 0.03	0.65 ± 0.14

Data represent mean ± standard deviation of triplicate determinants (*n* = 3). The yield (written in bolded text) represents the dry weight of the corresponding fraction compared to the dry weight of the algae.

## Data Availability

The data presented in this study are available on request from the corresponding author.
